# The Protective Efficacy of a SARS-CoV-2 Vaccine Candidate B.1.351V against Several Variant Challenges in K18-hACE2 Mice

**DOI:** 10.3390/vaccines12070742

**Published:** 2024-07-03

**Authors:** Jie Yang, Huifen Fan, Anna Yang, Wenhui Wang, Xin Wan, Fengjie Lin, Dongsheng Yang, Jie Wu, Kaiwen Wang, Wei Li, Qian Cai, Lei You, Deqin Pang, Jia Lu, Changfu Guo, Jinrong Shi, Yan Sun, Xinguo Li, Kai Duan, Shuo Shen, Shengli Meng, Jing Guo, Zejun Wang

**Affiliations:** 1 Wuhan Institute of Biological Products Co., Ltd., Wuhan 430207, China; 13163255896@163.com (J.Y.); qingdaoaoyu@163.com (H.F.); 2011302290006@whu.edu.cn (A.Y.); wangwenhui583@163.com (W.W.); wanxin130061@163.com (X.W.); linmumu19930221@163.com (F.L.); 18086496981@163.com (D.Y.); 13419684558@163.com (J.W.); kevin30596@163.com (K.W.); qq420789726@126.com (W.L.); caiqian555@163.com (Q.C.); 15327191982@163.com (L.Y.); 13387652736@163.com (D.P.); lujia1@sinopharm.com (J.L.); 13476229756@163.com (C.G.); shijinrong@sinopharm.com (J.S.); lixinguo@sinopharm.com (X.L.); duankai@sinopharm.com (K.D.); shenshuo1@sinopharm.com (S.S.); mengshengli@sinopharm.com (S.M.); 2 National Engineering Technology Research Center for Combined Vaccines, Wuhan 430207, China; 3 National Key Laboratory for Novel Vaccines Research and Development of Emerging Infectious Diseases, Wuhan 430207, China; 4 Hubei Province Vaccine Technology Innovation Center, Wuhan 430207, China; 5 Wuhan Institute for Neuroscience and Neuroengineering, South-Central University for Nationalities, Wuhan 430074, China; sunyan198112@mail.scuec.edu.cn

**Keywords:** SARS-CoV-2 variants, vaccine, immunogenicity, protection

## Abstract

The emergence of SARS-CoV-2 variants of concern (VOCs) with increased transmissibility and partial resistance to neutralization by antibodies has been observed globally. There is an urgent need for an effective vaccine to combat these variants. Our study demonstrated that the B.1.351 variant inactivated vaccine candidate (B.1.351V) generated strong binding and neutralizing antibody responses in BALB/c mice against the B.1.351 virus and other SARS-CoV-2 variants after two doses within 28 days. Immunized K18-hACE2 mice also exhibited elevated levels of live virus-neutralizing antibodies against various SARS-CoV-2 viruses. Following infection with these viruses, K18-hACE2 mice displayed a stable body weight, a high survival rate, minimal virus copies in lung tissue, and no lung damage compared to the control group. These findings indicate that B.1.351V offered protection against infection with multiple SARS-CoV-2 variants in mice, providing insights for the development of a vaccine targeting SARS-CoV-2 VOCs for human use.

## 1. Introduction

Since its discovery in late 2019, SARS-CoV-2 has caused a global pandemic with over 760 million infections and more than 6.9 million deaths worldwide as of 30 April 2024 (https://covid19.who.int/, accessed on 30 April 2024). Genetic mutations in SARS-CoV-2 have led to the emergence of variants of concern that exhibit higher transmissibility, immune evasion, and disease severity compared to the original strain [1,2,3,4]. Vaccination stands out as a cost-effective strategy to manage the pandemic. Currently, authorized vaccines for COVID-19 encompass mRNA vaccines, adenoviral-vectored vaccines, recombinant subunit vaccines, and purified inactivated vaccines, all of which have demonstrated positive efficacy in pre-clinical and clinical studies [5,6,7,8,9,10,11,12,13]. Purified inactivated virus (PIV) vaccines have shown protection against SARS-CoV-2 infection and disease in preclinical studies involving mouse and rhesus monkey models, eliciting robust specific IgG and neutralizing antibody responses [14]. Furthermore, phase 1/2 and 3 clinical trials in adults aged 18 years and older have demonstrated that PIV vaccines also induce high levels of T cell responses [15,16,17,18,19,20], thus leading to the emergency use authorization of PIV vaccines in 2020.

The emergence of new SARS-CoV-2 variants of concern, such as Alpha/B.1.1.7 in the United Kingdom [1,21], Gamma/P.1 in Brazil and Japan [22], Beta/B.1.351 in South Africa [2], Delta/B.1.617.2 in India [23], and Omicron/B.1.1.529 in South Africa and Botswana in November 2021 [24], has raised worries about their potential to enhance transmissibility and resistance to neutralizing antibodies from ancestral virus exposure or vaccination. Studies have indicated decreased effectiveness of the Oxford–AstraZeneca and Novavax vaccines in trials conducted in South Africa [25,26]. Furthermore, the levels of neutralizing antibodies produced by vaccination against the most antigenically diverse VOCs are often significantly lower than those against the original SARS-CoV-2 strain [27,28,29,30,31]. This has led to concerns that current vaccines based on the original SARS-CoV-2 strain may have reduced efficacy against certain VOCs [27,32,33].

As of April 2024, there are currently 356 SARS-CoV-2 vaccine candidates, with 138 in clinical testing (https://vac-lshtm.shinyapps.io/ncov_vaccine_landscape/, accessed on 30 April 2024). The majority of these candidates target the S protein and fall into five categories: DNA vaccines, mRNA vaccines, vectored vaccines, inactivated vaccines, and protein subunit vaccines. A total of 34 vaccines have been authorized for use in various countries, all aimed at inducing cellular and humoral immune responses against the original SARS-CoV-2 strain identified in Wuhan in January 2020 [23]. Concerns have been raised about the efficacy of these vaccines against variant viruses, as studies have indicated a reduction in effectiveness against mild to moderate disease in regions where variant strains are prevalent [33,34,35]. Previous research has emphasized that vaccines designed to target particular strains, such as XBB.1.5, BA.4, and BA.5, exhibit higher levels of neutralizing antibodies against the current viral strains, surpassing the original prototype vaccines [36,37,38]. This strong evidence underscores the essential requirement to consistently enhance and modify the formulation of current vaccines to ensure their efficacy against changing viral strains.

This study evaluated the immunogenicity of B.1.351V in BALB/c mice and demonstrated its effectiveness in providing protection against SARS-CoV-2 and other variants in K18-hACE2 mice. The results showed that B.1.351V induced strong immunity against both the original virus and the Beta and the Delta variant, and in BALB/c mice. K18-hACE2 mice vaccinated with B.1.351V and then exposed to a high dose of SARS-CoV-2 and two variant strains had lower viral loads in their lungs and fewer lung lesions compared to unvaccinated mice. This study successfully established a B.1.351 infection model in K18-hACE2 mice and evaluated the protective effect of the B.1.351 vaccine, providing valuable insights and guidance for the development of vaccines targeting mutant strains.

## 2. Materials and Methods

### 2.1. Ethical Approval

All animal studies were conducted at Wuhan Institute of Biological Products Co., Ltd.’s (WIBP) animal laboratory center, using 6~8-week-old female BALB/c mice and 4~5-week-old male K18-hACE2 transgenic mice. The research was granted ethical clearance under the approval number WIBP-AII 382021002. The SARS-CoV-2 infection experiments were approved at the BSL3 laboratory in WIBP. Throughout the experiments, strict adherence was maintained to the guidelines set forth in the Guide for the Care and Use of Laboratory Animals, as well as international standards and regulations concerning the welfare of animals used in research.

### 2.2. Cells and Viruses

Vero E6 cells were obtained from ATCC and Vero cells from the WIBP cell bank. All the cells were propagated in Dulbecco’s Modified Eagle’s Medium (Gibco, Waltham, MA, USA), which was supplemented with 10% fetal bovine serum (FBS), 100 mg/mL streptomycin, and 100 IU of penicillin (Gibco, Waltham, MA, USA) and cultured at 37 °C in a 5% CO_2_ incubator (Thermo Scientific, Waltham, MA, USA). The virus, named 501Y.V2, was isolated from a COVID-19 patient from abroad and was transported to WIBP under the coordination of the Health Commission of Hubei Province. The identity of the virus was confirmed by metagenomic sequencing. Briefly, the isolated 501Y.V2 virus underwent whole-genome sequencing and was compared with several B.1.351 genome sequences available in the NCBI database, demonstrating a homology of over 99%. When compared to the S gene of the WIV04 strain, three distinctive mutation sites of B.1.351 were identified: K417N, E484K, and N501Y. Sequence analysis definitively classified this virus as a B.1.351 variant.

### 2.3. Virus Titration

The virus titer was determined using a micro-cytopathic effect assay. The virus was serially diluted 10-fold and then mixed with 2 × 10^4^ Vero cells before being plated on 96-well culture plates. Following incubation for 3 to 5 days in a 5% CO_2_ incubator at 37℃, the cells were examined for the presence of a cytopathic effect (CPE) under a microscope. Titers for SARS-CoV-2 were determined through a 50% cell culture infective dose (CCID_50_) using the Spearman–Karber method.

### 2.4. Preparation of B.1.351V

The preparation of B.1.351V was conducted similarly to that of SARS-CoV-2 as outlined in our previous studies [16,18]. Initially, the virus was cultured in Vero cells. Then, the resulting supernatant was subsequently treated with β-propiolactone at a dilution of 1:4000 to inactivate the virus. After this step, the mixture was clarified to remove any cellular debris and then subjected to ultra-filtration to further purify the viral particles. Following these clarification steps, a second round of β-propiolactone inactivation was carried out. Subsequently, the inactivated virus harvest underwent purification via gel chromatography and ion-exchange chromatography. Finally, the purified virus harvest was combined with aluminum hydroxide (Croda, Frederikssund, Denmark, Cat No. 85643) to create vaccine candidates designated as B.1.351V. All manufacturing processes were carried out in GMP-compliant facilities and thoroughly documented.

### 2.5. Sodium Dodecyl Sulfate–Polyacrylamide Gel Electrophoresis (SDS-PAGE) and Western Blotting (WB)

The purified vaccine stock was used for SDS-PAGE and Western blotting analysis. Initially, total proteins were separated on 4–20% gradient polyacrylamide gels and migrated at a constant voltage of 100 V. After electrophoresis was completed, the gels were stained with Coomassie Blue (Sangon Biotech, Shanghai, China). At the same time, the proteins from the gels were transferred onto NC membranes for Western blotting analysis using different polyclonal antibodies. The gels and membranes were analyzed using Amersham Image Quant 800 (Cytiva, Marlborough, MA, USA).

### 2.6. Immunization and Infection

Two vaccines, WIV04V (purified from prototype WIV04 virus harvest) and B.1.351V (purified from 501Y.V2 virus harvest), were used for immunizing the animals. BALB/c mice were immunized with WIV04V and B.1.351V via intraperitoneal injection on day 0 and day 14. Blood samples were collected from the orbit on day 28 after the initial immunization. K18-hACE2 transgenic mice were divided into 6 groups, each containing 5 mice, including a B.1.351V group and an Al(OH)_3_ adjuvant group. These mice received two intraperitoneal injections on day 0 and day 14 with B.1.351V. Blood samples were collected from the orbit on day 25 after the first immunization. On day 28, the mice were intranasally infected with 200 CCID_50_ of WIV04, B.1.351, or B.1.617.2 per mouse under ABSL-3 containment conditions. The animals were monitored daily for any clinical signs of disease and weighed daily post challenge. On day 6 after virus infection, the mice were euthanized by CO_2_ asphyxiation, and lung tissues were collected for viral load detection and pathological analysis.

### 2.7. Enzyme-Linked Immunosorbent Assay (ELISA)

The antigen-specific IgG antibodies present in the serum of immunized mice were quantified using ELISA. The process began by coating 96-well plates with the vaccine stock solution at a dilution of 1 µg/mL in 100 µL of coating buffer per well and allowing them to incubate at 4 °C overnight. The following day, all wells were blocked with 200 µL of 0.01 M PBS containing 1% bovine serum albumin (BSA), and incubated at 37 °C for one hour to prevent non-specific binding. Subsequently, the wells were treated with a series of 10-fold dilutions of the serum samples and incubated for one hour at room temperature. After incubation, horseradish peroxidase (HRP)-conjugated anti-mouse IgG antibodies (Boster Biological Technology, Wuhan, China) were added at a dilution of 1:5000 and allowed to incubate for another hour. Between each step, the wells were thoroughly washed four times with PBS containing 0.05% Tween-20 (PBST) to remove any unbound material. Then, 100 μL of the TMB substrate (3,3′,5,5′-tetramethylbenzidine) was added to each well and incubated for 15–20 min at room temperature. The reaction was stopped by the addition of 100 μL of 2M sulfuric acid (H_2_SO_4_), and the optical density (OD) was measured at dual wavelengths of 450 nm and 630 nm using an ELISA plate reader (BioTek, Winooski, VT, USA).

### 2.8. Pseudovirus Neutralization Assays

The neutralizing activity of sera against the original SARS-CoV-2 (WVI04), Beta (B.1.351), Gamma (P.1), and Delta (B.1.617.2) was assessed using pseudovirus neutralization assays [39]. Serum samples were heat-inactivated at 56 °C for 30 min and then serially diluted 3-fold with DMEM. These diluted sera were mixed with an equal volume of pseudovirus in a 96-well plate. Negative and positive controls were included for comparison. The mixture was incubated for 1 h at 37 °C with 5% CO_2_. Huh-7 cells (2 × 10^4^ cells per well) were then added to all wells and incubated for 24 h under the same conditions. After incubation, luminescence was determined by a Britelite plus Reporter Gene Assay System (PerkinElmer, Waltham, MA, USA). Finally, the IC_50_ value was calculated by the highest dilution of the serum sample wells where the fluorescence units reduced by 50% comparing to the virus control wells.

### 2.9. Authentic Neutralization Assays

The neutralizing activity of sera against the original SARS-CoV-2 (WIV04), Beta (B.1.351), and Delta (B.1.617.2) was assessed using authentic virus microneutralization assays conducted following established protocols [20]. Serum samples were heated for inactivation at 56 °C for 30 min. After an initial 8-fold dilution, further 10-fold serial dilutions were prepared in maintenance medium and incubated with 100 CCID_50_ virus at 37 °C for 2 hours. The resulting mixture was then added to monolayer Vero E6 cells in a 96-well plate. Then, the plates were placed in a 5% CO_2_–air incubator at 37 °C for 5 to 7 days. The neutralizing titer was determined by identifying the highest dilution of serum that still caused a 50% decrease in cytopathic effects (CPE), with the titer being expressed as the reciprocal of this dilution.

### 2.10. Virus Load in Lung Tissues

The virus load in lung tissues was analyzed by quantitative real-time PCR (qRT-PCR) [40]. First, the lung tissues were lysed in RLT buffer (Qiagen, Germantown, MD, USA). Then, the RNA was extracted with a Qiagen RNeasy Mini kit (Qiagen, Germantown, MD, USA). qRT-PCR was employed to measure viral RNA levels with primers and probes, which were specific to the SARS-CoV-2 N gene and ORF gene. Finally, the viral genome copies were determined by correlating the obtained qRT-PCR data with a standard curve. This curve was established using a quantified RNA extracted from SARS-CoV-2 samples that had been previously titrated.

### 2.11. Lung Tissue Histopathology

Lung tissues were fixed in situ with 10% neutral buffered formalin. Once fixed, these tissues were carefully extracted from the thoracic cavity. Subsequently, the lung specimens were encased in paraffin blocks to provide support and facilitate sectioning. Thin sections were then prepared and subjected to staining using hematoxylin and eosin (H&E) by Histoserv. Histopathological analysis was conducted on one slide per mouse, with five mice per immunization group. The slides were examined using an Olympus BX41 microscope, and photomicrographs were captured using an Olympus DP71 digital camera (Olympus Corporation, Tokyo, Japan) and Olympus Cellsens 2.1 software.

### 2.12. Statistical Analysis

Geometric Mean Titers (GMT) of binding and neutralizing antibody titers were calculated using Prism 9.0 software (GraphPad Software, San Diego, CA, USA). The IC_50_ values for pseudovirus neutralization titers were determined through non-linear regression analysis in GraphPad Prism 9.0. A Mann–Whitney test was used to determine the significance of differences. Significance was indicated when the value of *P* < 0.05.

## 3. Results

### 3.1. Characterization of Purified B.1.351 Virions

**Figure 1:**
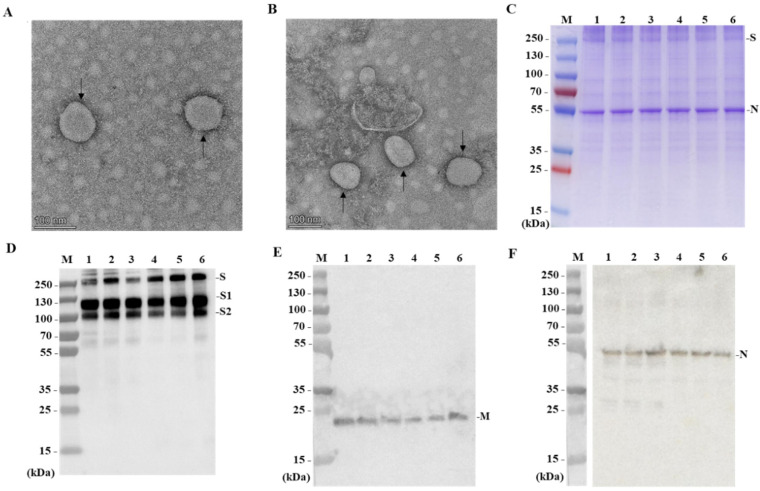
Analysis of purified B.1.351V virions. (**A**,**B**) The negatively stained, inactivated virion of WIV04 and B.1.351 visualized by TEM. The spike (S) glycoproteins on the viral envelope are indicated by arrowheads. The scale bar was set at 100 nm with a magnification of ×50,000. (**C**) SDS-PAGE of the purified, inactivated B.1.351 strain bulk. Proteins were separated on a 4~20% polyacrylamide gel and stained with Coomassie blue. (**D**–**F**) Western blotting of the purified, inactivated B.1.351 strain bulk. Viral proteins were analyzed by Western blotting with rabbit polyclonal antibodies against spike protein (S), membrane intrinsic protein (M), and nuclear protein (N). Lanes 1, 2, and 3 are three lots of the B.1.351 strain bulk; lanes 4, 5, and 6 are three lots of the prototype strain (WIV04) bulk. Molecular weight markers (kDa) are indicated on the left and viral structural proteins on the right.

The morphology of the purified B.1.351 virions was confirmed through transmission electron microscope (TEM) analysis using negative staining. The results indicated that the virions displayed spherical and pleomorphic shapes under TEM, with diameters ranging from 100 to 150 nm (Figure 1A,B). Additionally, the TEM images revealed spike proteins present on the envelope surface, suggesting that the native conformation of the spikes was maintained.

Proteins from purified virions were separated using 4~20% SDS-PAGE and stained with Coomassie Brilliant Blue R-250. The SDS-PAGE results showed no significant differences in the structural protein composition and location between the B.1.351 strain and the prototype strain (Figure 1C). These proteins were further confirmed by Western Blotting using rabbit polyclonal antibodies against spike protein (S) (Figure 1D), membrane intrinsic protein (M) (Figure 1E), and nuclear protein (N) (Figure 1F). This indicates that the major structural proteins of the B.1.351 strain were in line with those of the prototype, including S protein, N protein, and M protein.

### 3.2. B.1.351V Induced Humoral Responses against Variants in BALB/c Mice

**Figure 2:**
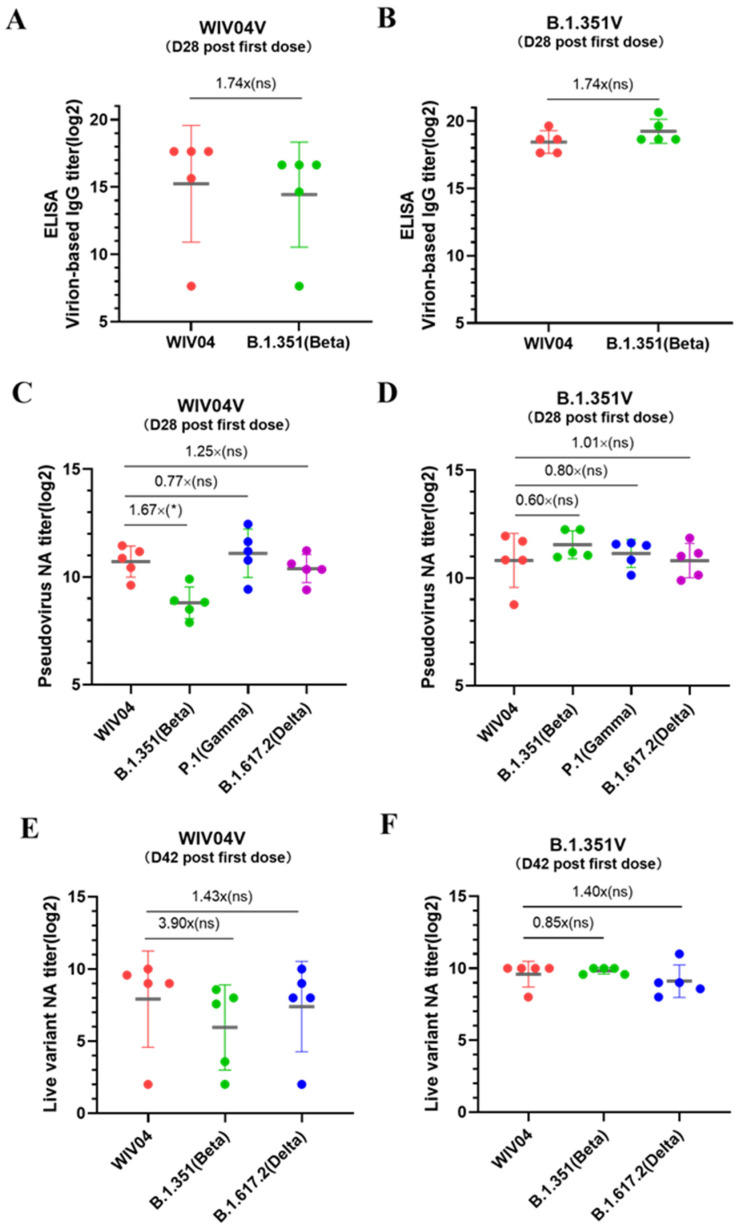
B.1.351V elicited robust binding and neutralizing antibody responses in BALB/c mice. (**A**,**B**) WIV04 virion-based IgG titers against WIV04 and B.1.351 on day 28 after the first dose. (**C**,**D**) Pseudovirus-neutralizing antibody titers (PNT) against WVI04, B.1.351, P.1 and B.1.617.2 variants on day 28 after the first dose. (**E**,**F**) Live virus-neutralizing antibody titers (LNT) against WVI04, B.1.351, and B.1.617.2 variants on day 42 after the first dose. *, *p* < 0.05; ns, not significant.

Virion-based binding antibody IgG geometric mean titers (GMTs) were measured 28 days after the first dose in mice that received WIBP-WVI04V. The titers were 38,802 and 22,288 against the WVI04 and B.1.351 strains, respectively, indicating a 1.74-fold reduction in IgG titers against B.1.351 compared to WVI04 (Figure 2A). In contrast, after the first dose of B.1.351V, the virion-based binding antibody IgG GMTs were 356,578 and 620,838 against the WVI04 and B.1.351 strains, respectively, showing a 1.74-fold increase in IgG titers against B.1.351 compared to WVI04 (Figure 2B). The binding antibodies in mice immunized with WIV04V and B.1.351V did not exhibit statistically significant differences in their reactivity against various viruses.

Using a VSV-based pseudovirus-neutralizing antibody (PNA) assay, we assessed the antibody responses induced by WIBP-WVI04V and WIBP-B.1.351V against various SARS-CoV-2 pseudovirus strains, including the WIV04, B.1.351, P.1, and B.1.617.2 variants. On day 28, mice that received the WIBP-WVI04 vaccine exhibited PNA-GMT values of 1650, 448, 2104, and 1341 against the WIV04, B.1.351, P.1, and B.1.617.2 strains, respectively. In comparison, a significant reduction in neutralizing antibody titers was observed for the B.1.351 variant, with a 1.67-fold decrease relative to the WIV04 strain (Figure 2C). Additionally, mice that received the B.1.351V vaccine demonstrated high and balanced PNA-GMT of 1802, 2982, 2250, and 1792 against the WIV04, B.1.351, and B.1.617.2 strains on day 28, indicating cross-neutralizing activity against different variants (Figure 2D). 

Live virus-neutralizing antibody (LNA) assays exhibited a similar trend to the PNA. In mice vaccinated with WIBP-WVI04V, the LNA GMTs at week 4 were 242, 62, and 169 against the WIV04, B.1.351, and B.1.617.2 strains, respectively. These assays demonstrated a more than 3.9-fold and a 1.42-fold decrease in neutralization titers against the B.1.351 and B.1.617.2 variants, respectively, compared to the original strain on day 42 (Figure 2E). However, the LNA did not demonstrate statistically significant differences in its neutralizing activity against the various viruses evaluated. The LNA GMTs in mice that received B.1.351V were 776, 913, and 555 against the WIV04, B.1.351, and B.1.617.2 strains, respectively, on day 42, indicating cross-neutralizing activity against different variants (Figure 2F).

### 3.3. Protection against Several SARS-CoV-2 Challenges in K18-hACE2 Mice 

**Figure 3:**
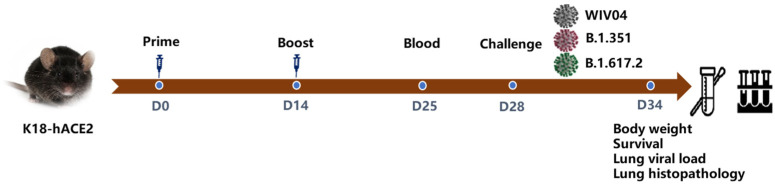
Overview of the immunization and challenge in K18-hACE2 mice. Mice (n = 5 per group) were immunized via the intraperitoneal route with WIBP-B.1.351V or adjuvant Al(OH)_3_, followed by a booster dose on day 14. Blood samples were collected on day 25. Mice were intranasally infected with 200 CCID_50_ of virus per mouse on day 28. Mice were sacrificed and lung tissues were collected on day 34.

The protection efficacy in K18-hACE2 mice was assessed as shown below (Figure 3). Mice were immunized with 5 μg of WIV04V (n = 5), B.1.351V (n = 5), or an adjuvant Al(OH)_3_ control (n = 5) via intraperitoneal injection on day 0, with a booster on day 14. Sera were collected from all vaccinated mice for antibody detection by day 25. On day 28, mice were infected with WIV04, B.1.351, and B.1.617.2 according to grouping, and on day 35, they were sacrificed for lung tissue collection.

**Figure 4:**
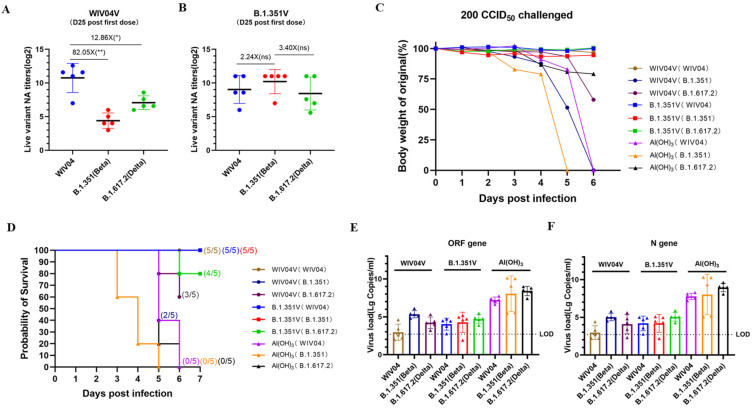
B.1.351V elicited protective efficacy in K18-hACE2 mice. (**A**,**B**) Live virus-neutralizing antibody (LNT) assays against the SARS-CoV-2 WVI04, B.1.351, and B.1.672.2 variants on day 25 after the first dose of the vaccine. (**C**,**D**) Weight loss and survival outcomes in K18-hACE2 transgenic mice after SARS-CoV-2 challenge. Weight changes and survival outcomes were monitored daily. (**E**,**F**) Viral loads (Lg copies/mL of ORF and N gene) in the lung tissue were measured after challenges with WIV04, B.1.351, and B.1.617.2 variants, with a limit of detection (LOD) of 102.7 copies per mL. **, *p* < 0.01; *, *p* < 0.05; ns, not significant.

Following the second dose of the WIV04V vaccine, the mice displayed robust neutralizing antibody titers, reaching 1723 against the WIV04 strain. In contrast, neutralizing antibody titers were comparatively lower against the B.1.351 and B.1.617.2 strains, at 21 and 134, respectively. The data revealed significant reductions in neutralizing antibody (NtAb) titers, with an 82.05-fold and a 12.86-fold decrease against the WIV04 strain when compared to the B.1.351 and B.1.617.2 strains, respectively (Figure 4A). Conversely, after the second dose of B.1.351V, all mice exhibited high neutralizing antibody titers of 524, 1176, and 346 against the WIV04, B.1.351, and B.1.617.2 strains, respectively. Notably, there was a 2.24-fold and 3.40-fold increase in NtAb titers against the B.1.351 strain in comparison to the WIV04 and B.1.617.2 strains. Importantly, the NtAb titers in mice immunized with B.1.351V did not exhibit any statistically significant differences when compared to those in other immunized groups (Figure 4B).

Weight loss and survival were evaluated in groups of five mice each following infection via the intranasal route with 200 CCID_50_/mouse of the WIV04, B.1.351, or B.1.617.2 strain. Al(OH)_3_-vaccinated mice infected with B.1.351 exhibited greater weight loss compared to those infected with WIV04 and B.1.617.2, showing an average weight loss of 7.9%, 21.8%, and 12.5% on day 4 post WIV04, B.1.351, or B.1.617.2 infection, respectively. Mice vaccinated with WIV04V and subsequently infected with the B.1.351 variant experienced more pronounced weight loss compared to those infected with the WIV04 or B.1.617.2 strain. Specifically, on day 5 post infection, the average weight loss was 1.7% for WIV04, 48.5% for B.1.351, and 6.7% for B.1.617.2, respectively. In contrast, mice immunized with B.1.351V experienced only slight weight loss following WIV04, B.1.351, or B.1.617.2 challenge throughout the observation period. On day 5 post infection, the average weight loss was 1.9%, 6.2%, and 0.9% for the WIV04, B.1.351, and B.1.617.2 strains, respectively (Figure 4C). In terms of survival, all control group mice succumbed to infection by day 6 post WIV04, B.1.351, or B.1.617.2 challenge. In the WIV04V-vaccinated group, survival was maintained until day 6 post WIV04 infection, with three fatalities observed after B.1.351 infection and two after B.1.617.2 infection. Conversely, mice immunized with B.1.351V survived until day 6 after WIV04, B.1.351, or B.1.617.2 infection, with the exception of one death in the B.1.617.2 challenge group (Figure 4D).

Viral loads in the lung tissues were estimated using qRT-PCR to detect copies of the viral ORF gene and N gene. Six days after infection with WIV04 and the variant viruses, mice in the Al(OH)_3_ group exhibited higher median copies, with 7.76, 7.63, and 8.93 Lg copies/mL of the ORF gene and 7.76, 7.63, and 8.93 Lg copies/mL of the N gene for the WIV04, B.1.351, and B.1.617.2 strains, respectively (Figure 4E,F). In comparison, the WIV04V-immunized mice showed median viral RNA copy numbers of 2.96, 5.34, and 4.22 Lg copies/mL for the ORF gene, and 2.96, 5.02, and 4.08 Lg copies/mL for the N gene, following infection with the WIV04, B.1.351, and B.1.617.2 strains, respectively. B.1.351V-immunized mice displayed median copies of 3.95, 4.02, and 4.68 Lg copies/mL of the ORF gene, and 4.09, 4.02, and 5.03 Lg copies/mL of the N gene, for the WIV04, B.1.351, and B.1.617.2 strains, respectively. These data indicated that the viral loads in the vaccine groups were approximately 1000-fold lower than those in the control mice.

### 3.4. Histopathology in Lungs

**Figure 5:**
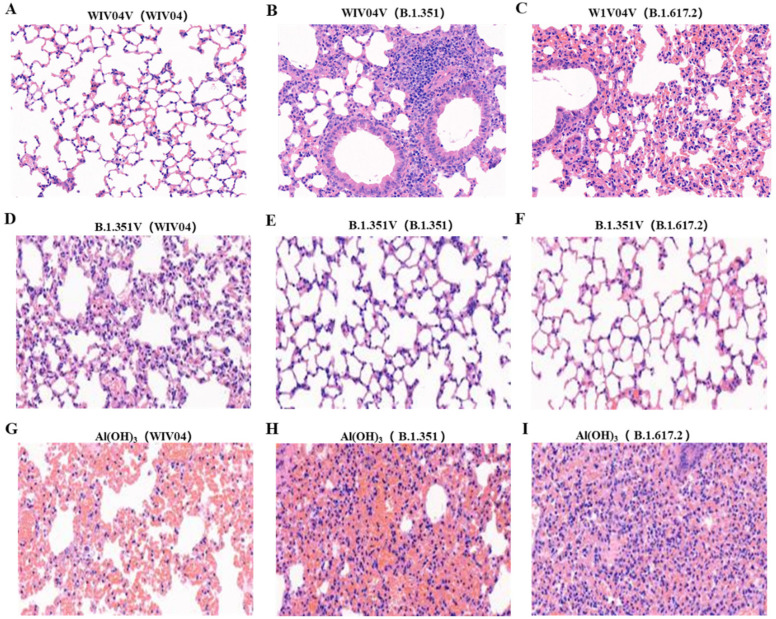
The results of the histopathological examinations for pathological changes in lung tissues. (**A**–**C**) Histopathology in lung tissues from WIV04V-vaccinated mice after WIV04, B.1.351, and B.1.617.2 challenge. (**D**–**F**) Histopathology in lung tissues from B.1.351V-vaccinated mice after WIV04, B.1.351, and B.1.617.2 challenge. (**G**–**I**) Histopathology in lung tissues from Al(OH)_3_-vaccinated mice after WIV04, B.1.351, and B.1.617.2 challenge. All images were captured at a magnification of 20×.

To assess the impact of WIV04V and B.1.351V vaccinations on lung injury following variant virus infection, lung tissues were examined for inflammatory pathology after 6 days of infection. The Al(OH)_3_ group mice displayed severe lung pathology, characterized by significant hemorrhage and consolidation areas in the alveoli, primarily concentrated around the trachea and blood vessels (Figure 5G–I). In contrast, mice vaccinated with WIV04V maintained a normal morphology and structure of their alveolar cells on day 6 post WIV04 challenge, albeit with extensive inflammatory cell infiltration and partial lung tissue consolidation following B.1.351 and B.1.617.2 challenges (Figure 5A–C). Notably, B.1.351V-vaccinated mice maintained a normal morphology and structure of their alveolar cells, with expanded marginal alveolar cells and adjacent alveoli merging into a large cystic cavity on day 6 post WIV04, B.1.617.2, or B.1.351 challenge (Figure 5D–F). These findings indicated that B.1.351V effectively prevented pulmonary damage following virus infection.

## 4. Discussion

The COVID-19 vaccines endorsed by various countries, whether approved or granted emergency use authorization, have played a crucial role in enhancing public health and serving as a lifeline for countless individuals globally. Despite this positive development, the SARS-CoV-2 virus responsible for COVID-19 continues to undergo evolution. The appearance of new strains, such as the Omicron variant, has resulted in notable increases in infection rates, underscoring the virus’s ability for rapid transmission and the persistent challenges it poses to global health [41,42].

The continuous emergence of SARS-CoV-2 VOCs highlights the need for updating current COVID-19 vaccines and developing vaccines that offer broader protection [43]. Numerous SARS-CoV-2 vaccines, including those based on the inactivated virus, mRNA, non-replicating adenovirus vectors, and protein-based vaccines, have been approved or authorized for emergency use. These vaccines have shown promising efficacy in preventing COVID-19 in phase 3 or phase 4 clinical trials. The varying effectiveness of different SARS-CoV-2 vaccine platforms may be influenced by factors such as vaccine design, the presence of SARS-CoV-2 variants, and the timing of vaccination [44,45]. However, the emergence of new variants of SARS-CoV-2 and its global spread have raised concerns about the potential decrease in protection offered by current COVID-19 vaccines against VOCs. 

The current vaccines have played a crucial role in reducing the most severe effects of COVID-19, such as hospitalizations and deaths. However, data from post-marketing surveillance studies suggest that the effectiveness of the initial vaccine series decreases over time, especially against newer viral strains like the Omicron variant [46,47]. While initial booster shots have been successful in restoring some level of immunity against severe outcomes and hospitalizations related to the Omicron variant, research shows that the effectiveness of these initial booster doses also diminishes over time [48,49,50]. The appearance of SARS-CoV-2 variants that could partially evade neutralizing antibodies presents a challenge to the effectiveness of existing COVID-19 vaccines. Our findings demonstrate that B.1.351V can induce cross-reactive humoral immune responses in BALB/c mice and K18-hACE2 transgenic mice, providing protection against SARS-CoV-2 WIV04, B.1.351, and B.1.617.2 challenges in K18-hACE2 transgenic mice. Vaccination with B.1.351V prevents pulmonary parenchyma, reduces lung damage, and significantly decreases viral replication in the lungs, suggesting that B.1.351V may reduce the risk of severe SARS-CoV-2-associated pathology in vaccinated K18-hACE2 transgenic mice. No specific adverse effects related to the vaccines were observed during the nonclinical safety evaluation. These results have important implications for the potential efficacy of the B.1.351 variant and the creation of future vaccine strategies against circulating variants of concern. This information is expected to guide the development of vaccines that can effectively combat the challenges presented by evolving SARS-CoV-2 variants.

Admittedly, there are limitations in our study. Firstly, we observed varying trends in antibody titers across different experimental groups, with some showing an increase and others a decrease. However, not all observed differences were statistically significant. This lack of significance is likely due to the limited sample size of the experimental animals and the considerable individual variability present within the study. Despite these limitations, it was noted that the antibody titers in all groups of B.1.351V were consistently higher than those in the WIV04V group. To enhance the reliability of future studies, increasing the number of animals may help to reduce the impact of individual differences, thereby providing more robust statistical outcomes. Secondly, SARS-CoV-2 mutated faster than vaccine development, resulting in the selection of B.1.351 antigens that may offer reduced protection against currently circulating strains (XBB.1.5 and JN.1). However, our research could potentially enhance our comprehension of VOCs, offering valuable insights for future vaccine design and development.

## 5. Conclusions

Our research findings indicate that the B.1.351V strain can elicit cross-reactive humoral immune responses in both BALB/c mice and K18-hACE2 transgenic mice, protecting against various SARS-CoV-2 strains, including WIV04, B.1.351, and B.1.617.2, in a K18-hACE2 transgenic mouse model. Vaccination with B.1.351V shows efficacy in preventing pulmonary parenchymal damage, reducing lung injury, and significantly inhibiting viral replication in the lungs. These results suggest that the B.1.351V vaccine may reduce the risk of severe pathology associated with SARS-CoV-2 infection in vaccinated K18-hACE2 transgenic mice. These findings have significant implications for the potential effectiveness of the B.1.351 variant and the development of future vaccine strategies targeting circulating variants of concern. This knowledge is expected to inform the design of vaccines that can effectively address the evolving challenges posed by SARS-CoV-2 variants.

## Data Availability

The original contributions presented in this study are included in the article; further inquiries can be directed to the corresponding authors.
